# Assessing the impact of MRI based diagnostics on pre-treatment disease classification and prognostic model performance in men diagnosed with new prostate cancer from an unscreened population

**DOI:** 10.1186/s12885-022-09955-w

**Published:** 2022-08-11

**Authors:** Artitaya Lophatananon, Matthew H. V. Byrne, Tristan Barrett, Anne Warren, Kenneth Muir, Ibifuro Dokubo, Fanos Georgiades, Mostafa Sheba, Lisa Bibby, Vincent J. Gnanapragasam

**Affiliations:** 1 Division of Population Health, Health Services Research & Primary Care Centre, University of Manchester, Manchester, UK; 2 Department of Urology, Cambridge University Hospitals NHS Foundation Trust, Cambridge, UK; 3 Department of Radiology, University of Cambridge, Cambridge, UK; 4 Department of Pathology, Cambridge University Hospitals NHS Foundation Trust, Cambridge, UK; 5 Division of Urology, Department of Surgery, University of Cambridge, Cambridge, UK; 6 Kasr Al Any School of Medicine, Cairo University, Giza, Egypt; 7 Cambridge Urology Translational Research and Clinical Trials Office, Addenbrooke’s Hospital, Cambridge Biomedical Campus, Cambridge, UK

**Keywords:** Prostate cancer, Prognostic groups, MRI Reclassification, Overall survival, Cancer specific survival

## Abstract

**Introduction:**

Pre-treatment risk and prognostic groups are the cornerstone for deciding management in non-metastatic prostate cancer. All however, were developed in the pre-MRI era. Here we compared categorisation of cancers using either only clinical parameters or with MRI enhanced information in men referred for suspected prostate cancer from an unscreened population.

**Patient and methods:**

Data from men referred from primary care to our diagnostic service and with both clinical (digital rectal examination [DRE] and systematic biopsies) and MRI enhanced attributes (MRI stage and combined systematic/targeted biopsies) were used for this study. Clinical vs MRI data were contrasted for clinico-pathological and risk group re-distribution using the European Association of Urology (EAU), American Urological Association (AUA) and UK National Institute for Health Care Excellence (NICE) Cambridge Prognostic Group (CPG) models. Differences were retrofitted to a population cohort with long-term prostate cancer mortality (PCM) outcomes to simulate impact on model performance. We further contrasted individualised overall survival (OS) predictions using the Predict Prostate algorithm.

**Results:**

Data from 370 men were included (median age 66y). Pre-biopsy MRI stage reassignments occurred in 7.8% (versus DRE). Image-guided biopsies increased Grade Group 2 and ≥ Grade Group 3 assignments in 2.7% and 2.9% respectively. The main change in risk groups was more high-risk cancers (6.2% increase in the EAU and AUA system, 4.3% increase in CPG4 and 1.9% CPG5). When extrapolated to a historical population-based cohort (*n* = 10,139) the redistribution resulted in generally lower concordance indices for PCM. The 5-tier NICE-CPG system outperformed the 4-tier AUA and 3-tier EAU models (C Index 0.70 versus 0.65 and 0.64). Using an individualised prognostic model, changes in predicted OS were small (median difference 1% and 2% at 10- and 15-years’ respectively). Similarly, estimated treatment survival benefit changes were minimal (1% at both 10- and 15-years’ time frame).

**Conclusion:**

MRI guided diagnostics does change pre-treatment risk groups assignments but the overall prognostic impact appears modest in men referred from unscreened populations. Particularly, when using more granular tiers or individualised prognostic models. Existing risk and prognostic models can continue to be used to counsel men about treatment option until long term survival outcomes are available.

**Supplementary Information:**

The online version contains supplementary material available at 10.1186/s12885-022-09955-w.

## Introduction

The use of Magnetic Resonance Imaging (MRI) to triage and inform biopsies has revolutionised prostate cancer diagnostics [1, 2]. It is now an indispensable part of the pathway and endorsed by numerous guidelines. The evidence is clear that it has a high negative predictive value in ruling out cancer. However, it is less clear whether its introduction will improve risk stratification and hence better inform prognostic models and survival outcomes. This is particularly important in the unscreened context where cancers are less likely to be detected at the indolent stage [3].

Systematic reviews have suggested that MRI does help detect more clinically significant cancers [4]. Other studies have reported better staging of tumours compared to clinical staging alone with radical prostatectomy as a standard. In contrast, a number of randomised controlled trials have not shown any difference in clinically significant detection rates compared to non-image-based pathways [5–8]. In addition, modelling studies have suggested that cancers detected by MRI (and missed by standard biopsies) may not have a significant lethal effect over time [9, 10]. Thus, the question of its role in impacting and improving survival remains debated and long-term data will take years to mature [10–13].

Risks and prognostic groupings are critical in the pre-treatment decision making process for informing the optimal management for men with newly diagnosed prostate cancer [1]. Current model systems however were developed in the pre-MRI era using clinical staging and systematic biopsies only. Despite this, they have been shown to have high predictive ability for cancer related mortality in large long-term cohort studies particularly using recent refinements in categorisation [14–16]. Because of the long natural history of the disease, it is as yet unknown how their performance may or may not be altered by MRI nor which models are less or more affected.

Herein we undertook a comparison of the categorisation of cancers detected in a contemporary cohort of patients using either only clinical parameters (examination and systematic biopsies) or with MRI based staging and targeted biopsies. Our goal was to assess to what extent MRI changed the distributions in risk and prognostic group assignment *before* a treatment is decided and to simulate the potential impact on risk and prognostic model performance. Our particular interest was the impact of MRI in the context of men who are not screened and hence without a high penetrance of early PSA testing.

## Patients and methods

### Cohort assembly

#### Study cohort

This was assembled from a database of men investigated for suspected prostate cancer by pre-biopsy MRI in our unit (2015–2021) and enrolled into an ethically approved study (REC 03/018, Cambridgeshire 2 Research ethics Committee, Cambridgeshire, UK). Informed consent for enrolment into this study was obtained from each participant. The study was also registered and approved by Cambridge University Hospitals NHS trust clinical audit department (registration number PRN8595). Patient recruitment, use of anonymised data and study method were approved by these bodies for this analysis. The standard protocol was a clinical assessment following a primary care referral, repeat examination and history and then MRI. In about a third of cases the MRI occurred before the clinic review and was available prior to the clinical examination. The key inclusion criteria were: available data on both clinical Digital Rectal Examination (DRE) stage performed in the diagnostic clinic and MRI stage, MRI lesion positivity and Likert score, as well as available details of histology from both systematic biopsies and, if a lesion was present, image-fusion guided targeted biopsies. Systematic biopsies included 3 cores each taken from the right and left side of the prostate and from base and apex respectively (12 cores in total). Targeted biopsies included 2–3 samples taken from the target site guided by the MRI using image guided fusion software as previously described [17]. For clinical staging the 2017 TNM classification (version 8) was used. For radiological staging we used the following method (i) Biopsy-proven MRI-invisible lesions are assigned radiological rT1 stage (ii) MRI-visible organ-confined lesions are assigned rT2 stage and Locally-advanced rT3 lesions were subclassified as rT3a or rT3b disease. There were no T4 cases in our series. Men with a negative MRI (Likert score 1–2) but other clinical suspicion (Prostate Specific Antigen density [PSAd] ≥ 0.15) or patient choice to have a biopsy underwent systematic biopsies only. Men were excluded if they had had a previous biopsy, pelvic metalwork interfering with MRI quality or no MRI, or if no biopsy was done after MRI. All men were referred based on primary care-based assessment and serendipitous PSA testing as screening programmes are not used in the UK. Only men with a diagnosis of non-metastatic prostate cancer were included in this study.

#### MRI Protocol

Patients underwent prostate MRI on a 1.5 T (approximately 15%) MR450 or 3.0 T (85%) Discovery MR750 HDx (GE Healthcare, Waukesha, USA) with a 16–32 channel surface phased array coil. The MRI protocol was fully PI-RADS compliant and has previously been described in detail [17, 18]. In brief, the protocol included axial T1, T2 in the axial (slice thickness 3 mm; gap 0–1 mm), sagittal and coronal planes, with functional diffusion-weighted (DWI) and dynamic contrast-enhanced (DCE) sequences. The axial T2, DWI and DCE sequences were matched in orientation, slice thickness and gap. Axial DWI was performed with slice thickness 3–4 mm; gap 0 mm with b-values: b-150, b-750, b-1,400 s/mm^2^ and additional 2,000 s/mm^2^ at 3 T, with automated apparent diffusion coefficient (ADC) maps calculated. Axial DCE was acquired following bolus injection of Gadobutrol (Gadovist, Schering AG), temporal resolution 7 s/10 s. MR Images were interpreted by one of four specialist uroradiologists with 6–13 years’ experience and considered experts based on the number of MRIs reported [19, 20]. MRI sequences were evaluated based on PI-RADS structured scoring criteria, with weighting applied for T2 and DWI scoring depending on location in the PZ or TZ [7]. An overall impression was then used to derive a Likert suspicion score, wherein 1) csPCa highly unlikely, 2) csPCa unlikely, 3) indeterminate, 4) csPCa likely, 5) csPCa highly likely [21].

#### Population cohort

To simulate the potential effect of MRI based diagnostics on risk model performance, we used data from a large population cohort previously published to develop the Cambridge Prognostic Group (CPG) model and for which ethical approval was not required as it based on fully anonymised data [22]. Briefly, primary prostate cancers (ICD-10 code C61) diagnosed in residents of the East of England Cancer Network area between January 1st 2000 and December 31st 2010 were registered by the Public Health England National Cancer Registration Service Eastern Office (NCRS[E]). Cases with any metastatic involvement (as documented by M stage disease and/or positive bone or CT scan) were excluded. The stage assigned to each tumour was an integrated TNM stage (fifth edition up to 2009 and seventh edition in 2010). Cause of death was classified as prostate cancer specific, other death or censored if alive. The median follow-up was 6.9 y for the primary cohort. Only cases with all components of diagnostic stage, primary and secondary grade, and presenting PSA (ng/ml) as well as data on follow-up and survival were included.

### Analysis

Clinical and MRI enhanced data from the study cohort was first analysed to compare clinic-pathological and risk group assignment between them (i.e., DRE clinical staging and histology from systematic biopsies only) versus MRI enhanced data (MRI radiological stage and histology from combined systematic and targeted biopsies). Comparisons were made in the percentage presentation by histological grade and stage distribution. Distribution of risk groups was compared using 3 model systems: European Association of Urology (EAU) 3-tier system [23], American Urological Association 4-tier system [24] and the recently described 5-tier Cambridge Prognostic Group system which is now adopted by the UK National Institute for Health and Care Excellence as the national standard for risk stratification [14, 22]. Of note the EAU and AUA models also use T2 substages in that T2c is assigned into the high-risk group. However, the 2017 (version 8) update to the TNM system have now abandoned the use of T2 substages due to a lack of evidence of clinical impact. As a result (and for simplicity) all men were only assigned as whole T sub-groups for all classification models. These differences were then retrofitted to the population cohort dataset with long-term survival outcomes. This was done to simulate any potential changes in risk group performance in prediction of prostate cancer mortality (PCM). For this the *stratarand* function (Stata, release 15, StataCorp, TX, USA.) was used. This function assigned mortality events to the refitted experimental groups in each stratum by assigning blocks of observations to a given outcome based on the percentage from the original cohort. An example on how this was done is shown in Supplementary methods M1 for illustration using the CPG model. Finally, to estimate the effect on individual predictions of overall survival (OS) and benefit of treatment, we derived contrasting individual predictions of outcome using the Predict Prostate (https://prostate.predict.nhs.uk) algorithm [25]. The mean and median differences in the overall cohort from these estimates were calculated and compared using the students T test. All methods were carried out in accordance with relevant guidelines and regulations.

## Results

### Clinical versus MRI-based disease characterisation in an MRI pre-biopsy cohort

**Fig. 1:**
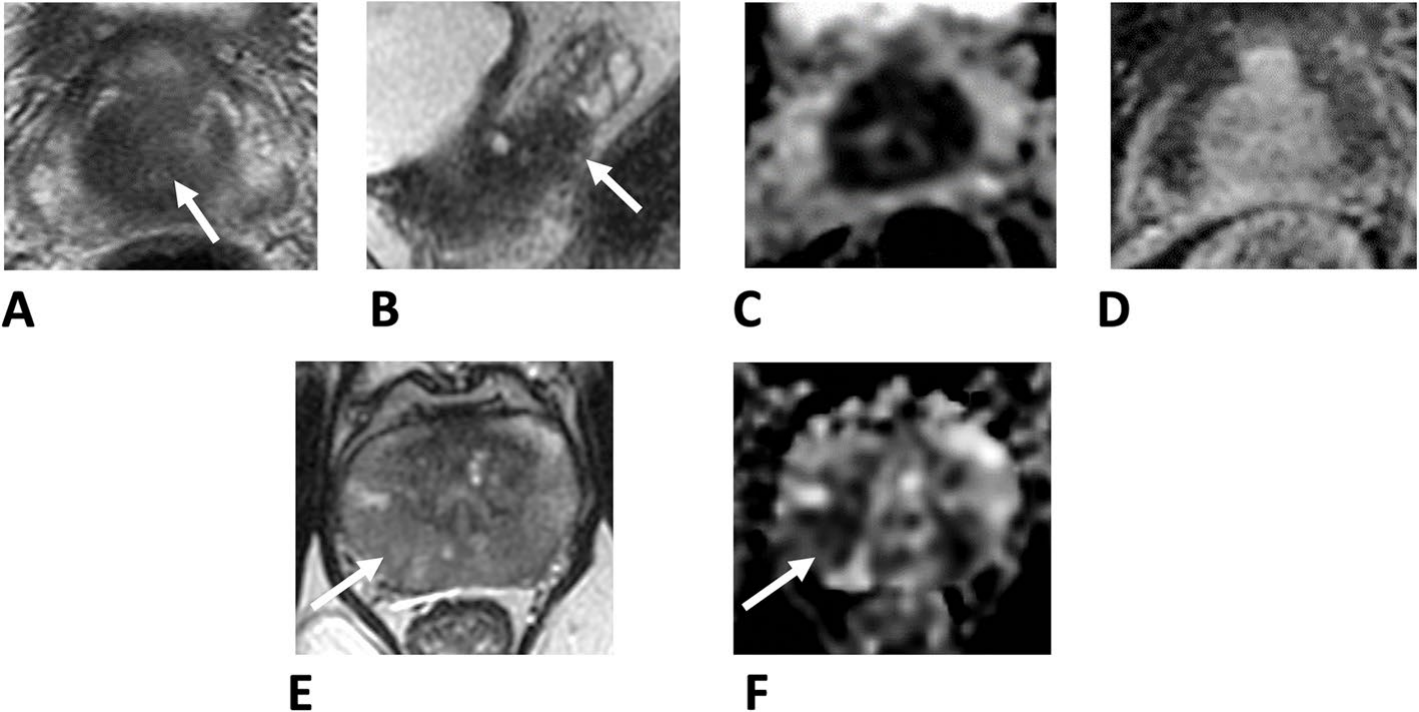
A-D. Exemplar case where MRI changed both clinical stage and biopsy grade: 66-year-old biopsy-naïve patient, PSA 19 ng/mL. A, B: T2 axial images show a large 29 × 22 mm lesion centred on the central zone (arrow in A), with clear seminal vesicle invasion seen on sagittal imaging (arrow in B). C, D: The lesion demonstrates marked restricted diffusion on ADC maps (C) and clear early enhancement on DCE (D). Target cores demonstrated Gleason 4 + 5 = 9 (Grade Group 5), systematic cores however showed right-sided Gleason grade 4 + 3 = 7 (Grade group 3). The location in the central zone make systematic cores less likely to sample the tumour core, with additional T3b disease shown on MRI. The prognostic group changed from CPG3 to CPG5. E–F Exemplar case where clinical and MRI based investigations concurred: 52-year-old biopsy naïve patient, PSA 16.4 ng/mL. A, B: bilaterally PZ lesions with low T2 signal (E) and matching restricted diffusion on ADC map (F), measuring up to 15 mm in the right mid PZ (arrows) and 13 mm in the left mid PZ. Right-side target cores demonstrated Gleason grade 3 + 4 = 7 (Grade Group 2) and left targets exhibited Gleason grade 3 + 3 = 6 (Grade Group 1), replacing approximately 79% of cores. Systematic biopsy showed Gleason grade 3 + 4 = 7 (Grade Group 2). The large size of the lesions and in the mid gland PZ posteriorly, ensured adequate sampling by systematic biopsy. The prognostic group was unchanged as CPG2. (CPG—NICE Cambridge Prognostic Group model)

The study cohort included 370 men with a median age of 66 (range 45-80y) and median PSA of 9 (range 1.2–108) (Table 1). 17/370 (4.5%) had a negative MRI and proceeded to biopsy for ongoing suspicion of cancer (high PSA density or patient preference). Of those with MRI lesions 29 were Likert 3, 79 were Likert 4 and 193 were Likert 5. Likert scores were not recorded in the remaining 52 MRI positive lesions. Table 2 shows the distribution of stage by DRE (digital rectal examination) and MRI as well as distribution of histological Grade Group by systematic biopsies only or a combination of systematic and targeted biopsies. MRI increased the disease stage assignment in 7.8% of patients compared to clinical DRE. In histological distributions there were minor changes between the overall Grade Group classification comparing systematic only versus targeted and systematic sampling. The main shift was fewer Grade Group 1 cases (-1.4%) and an increase in Grade Group 2 (2.7%). 11 (2.9%) further men were reassigned to ≥ Grade Group 3. In 16 (4.3%) cases the systematic biopsies were benign but targeted sampling had identified cancer. In all, grade shift was identified in 5.7% of cases. The changes above resulted in relatively small changes in overall risk group classification (Table 2). The main change was an increased proportion of high-risk cancers (6.2%) in the EAU and AUA risk categorisations primarily due to the stage shift from MRI re-classification. The CPG system exhibited this in more granular detail with a 4.3% increase in CPG4 (high risk) and 1.9% increase in CPG5 (very high risk) assignments. Exemplar cases from our series where MRI did and did not change risk assignment are shown and detailed in Fig. 1.

**Table 1:** Demographic and diagnostic features of the study cohort used in this analysis. All men were diagnosed based on image-based biopsies and had both clinical and MRI based staging and biopsies

	Study cohort *n* = 370	
Age (years)	mean	66
	median	67
	range	(45–80)
PSA (ng/ml)	mean	13
	median	9
	range	(1.2–108)
Prostate volume (mls)	mean	47
	median	41
	range	(17.9–191)
MRI LIKERT SCORE	1–2	17
	3	29
	4	79
	5	193
	Not stated	52
No of biopsy cores taken	Mean	17
	Median	15
Biopsy cores with cancer	Mean	7
	Median	6

**Table 2:** Comparative clinico-pathological characteristics and risk group assignments in the study cohort between clinical based characterisation (DRE + systematic sampling only) versus MRI based characterisation (MRI staging and combined MRI targeted and systematic sampling) in MRI-prebiopsy cohort. *no T4 cases in this cohort, see methods for how stage was assigned for clinical and MRI assignment. European Association of Urology (EAU), American Urological Association, Cambridge Prognostic Group (CPG)

	**Clinical stage and systematic biopsies**		**MRI stage and target systematic biopsies**		**Comparison**
	**n**	**Percentage (%)**	**n**	**Percentage (%)**	**(%)**
Stage ^*^					
T1-T2	263	71.1	234	63.2	-7.8
T3	107	28.9	136	36.8	7.8
**Grade Group**					
1	101	27.3	96	25.9	-1.4
2	123	33.2	133	35.9	2.7
3	60	16.2	63	17.0	0.8
4	13	3.5	17	4.6	1.1
5	57	15.4	61	16.5	1.1
Benign	16	4.3	-	-	-4.3
**EAU (Cancers only)**	(*n* = 354)		(*n* = 370)		
Low risk	67	18.9	66	17.8	-1.1
Intermediate risk	155	43.8	143	38.7	-5.1
High	132	37.3	161	43.5	6.2
**AUA (Cancers only)**					
Low risk	67	18.9	66	17.8	-1.1
Favourable intermediate risk	86	24.3	78	21.1	-3.2
Unfavourable intermediate risk	69	19.5	65	17.6	-1.9
High risk	132	37.3	161	43.5	6.2
**CPG (Cancers only)**					
1	67	18.9	66	17.8	-1.1
2	86	24.3	78	21.1	-3.2
3	69	19.5	65	17.6	-1.9
4	64	18.1	83	22.4	4.3
5	68	19.2	78	21.1	1.9

### Impact on prostate cancer mortality prognostic models

**Fig. 2:**
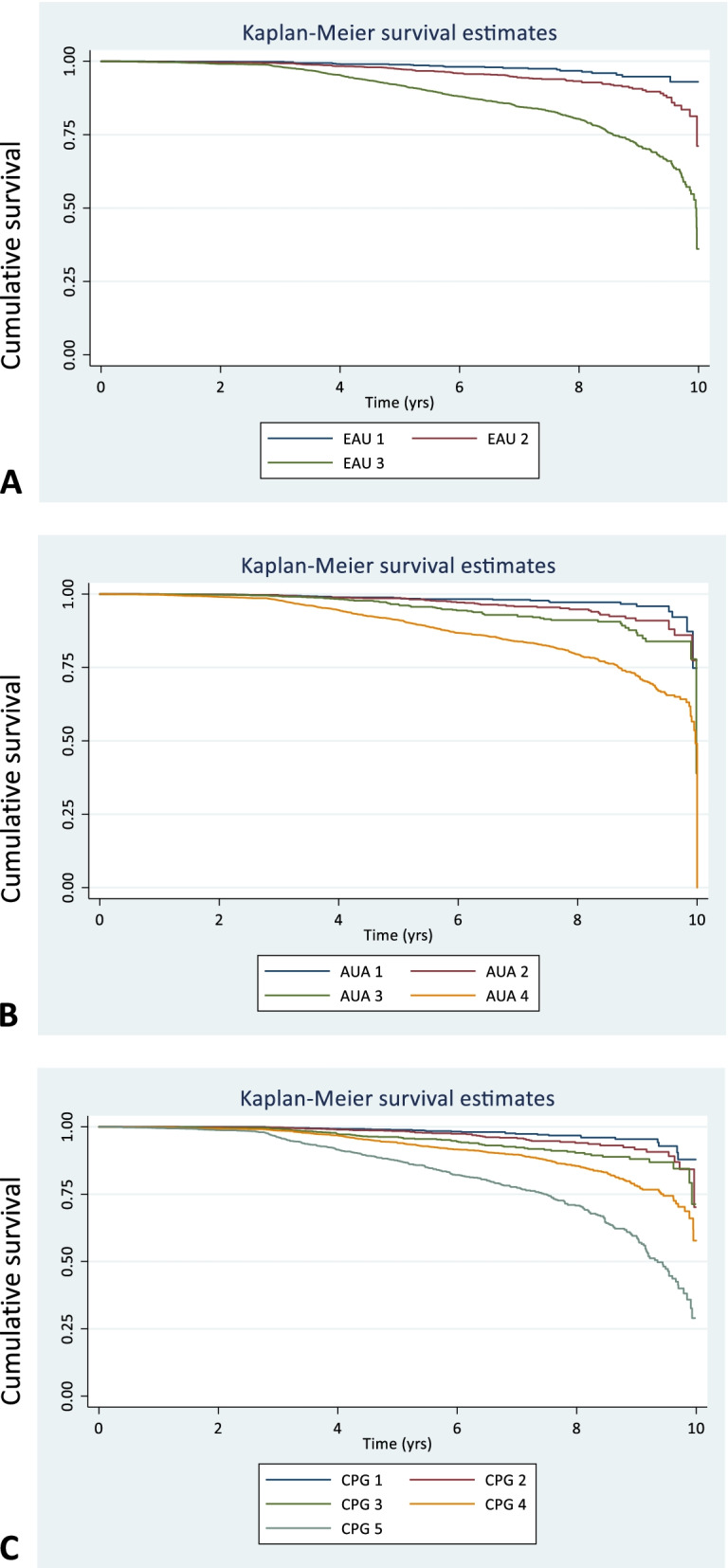
Simulated Kaplan–Meier curves for prostate-cancer-specific survival based on applying MRI adjustments to a previously described population cohort dataset of 10,139 men with long-term survival outcomes [24] A. European Association of Urology (EAU) 3-tier risk model B. American Urological Association (AUA) 4-tier risk model C. UK National Institute for Health and Care Excellence – Cambridge Prognostic Group 5-tier (CPG) prognostic model

To understand how MRI reclassification might impact risk model performance for PCM, we simulated a scenario where the percentage differences seen above was applied to adjust risk allocations in a previously reported large cohort with long term (10 year) survival data [14]. The final cohort comprised 10,139 individuals, with 789 prostate cancer deaths. Table 3 shows the distributions by different risk groupings (EAU, AUA and CPG) in the original and re-distributed simulated model. The method we used then assigned the proportions of patients alive and deaths in the original model to the redistributed groups and then re-tested model discrimination as a whole. In all 3 models the redistributions in the new simulated risk grouping retained different hazard ratios for prostate cancer mortality (Table 3). Overall discrimination was maintained in all groups although predictive power was altered in the simulation with the redistributed cohorts resulting in generally lower C indices. The best model performance was, in order, the CPG system (C-index 0.70, CI 0.67–0.71), AUA (0.65, CI 0.64–0.67) and EAU (0.64, CI 0.63–0.66) (Table 3). Kaplan–Meier curves illustrating the discrimination between risk strata is shown in Fig. 2.

**Table 3:** Comparative prognostic model performance in a large population cohort (n = 10,139) between the original risk group allocation and a simulated reassignment of risk allocation by applying the observed differences from Table 2 between clinical based characterisation (DRE + systematic sampling only) versus MRI based characterisation (MRI staging and combined MRI targeted and systematic sampling). European Association of Urology (EAU), American Urological Association, Cambridge Prognostic Group (CPG). *Death due only to prostate cancer

	**Original allocation**					**Redistributed allocation**				
**Risk model**	**Alive**	Dead*	**Hazard ratio (CI)**	***p* value**	**C-index (CI)**	**Alive**	Dead*	**Hazard ratio (CI)**	***p* value**	**C-index (95% CI)**
**EAU**										
Low	1707	33	Reference	–	0.69 (0.68–0.71)	1596	31	Reference	–	0.64 (0.63–0.66)
Intermediate	3560	155	2.7 (1.9–4.1)	< 0.0001		3070	134	2.1 (1.4–3.1)	< 0.0001	
High	4083	601	9.0 (6.3–12.8)	< 0.0001		4627	681	6.2 (4.3–8.9)	< 0.0001	
**AUA**										
Low	1707	33	Reference		0.71 (0.70–0.72)	1596	31	Reference		0.65 (0.64–0.67)
Favourable Intermediate	2015	63	1.9 (1.2–2.8)	0.004		1700	53	1.55 (1.0–2.4)	0.05	
Un-favourable Intermediate	1545	92	3.9 (2.7–5.9)	< 0.0001		1370	81	2.8 (1.8–4.2)	< 0.0001	
High	4083	601	9.0 (6.4–12.8)	< 0.0001		4627	681	6.3 (4.4–9.0)	< 0.0001	
**CPG**										
1	1707	33	Reference		0.75 (0.74–0.77)	1596	31	Reference		0.70 (0.67–0.71)
2	2015	63	1.9 (1.2–2.9)	0.004		1700	53	1.6 (0.99–2.4)	0.05	
3	1545	92	4.90 (2.7–6.0)	< 0.0001		1370	81	2.8 (1.8–4.2)	< 0.0001	
4	2784	268	5.6 (3.9–8.1)	< 0.0001		3177	306	4.3 (3.0–6.3)	< 0.0001	
5	1299	333	18.4 (12.9–26.4)	< 0.0001		1452	373	9.8 (6.8–14.1)	< 0.0001

### Impact on individualised overall survival estimates and treatment benefit

We next tested how MRI reclassification might alter individualised predictions of overall survival outcomes when other cause mortality was considered. To do this we used the validated Predict Prostate tool [25, 26]. Overall survival outcomes and estimated differences in treatment benefit were calculated for each individual using clinical only or MRI enhanced clinic-pathological cancer features (*n* = 354) (Table 4). No changes in overall survival estimates were seen in 81.3% and 78.8% of cases at 10 and 15 years respectively (Table 4). Amongst the cases that did have a change in predicted outcome, the median difference in overall survival was 1% and 2% at 10 and 15 years respectively (Table 4). Only 6/354 (1.6%) and 8/354 (2.3%) of patients had a ≥ 10% difference in predicted 10 year and 15-year overall survival estimates. Estimated treatment benefit was unchanged for the majority of cases (85.4% and 81.3% at 10 and 15 years respectively). The median difference in treatment benefit was 1% at both 10- and 15-years’ time frame. Only 5/354 (1.4%) and 2/354 (0.6%) of patients had a ≥ 5% difference in estimated 10- and 15-year treatment benefit.

**Table 4:** Comparison of individualised prognostics predictions of 10 and 15 year overall survival and treatment benefit outcomes (radical treatment versus conservative management) based on the Predict Prostate algorithm (https://prostate.predict.nhs.uk) using either clinical based characterisation (DRE + systematic sampling only) versus MRI based characterisation (MRI staging and combined MRI targeted and systematic sampling)

**10-year survival outcome**	**No change in predicted outcome**	**288 (81.3%)**
**(*n* = 354)**		
	**Number with a change**	**66 (18.7%)**
	Range of change in survival	-20% to 9%
	Mean change in survival	-1.86%
	Median change in survival	-1.0%
**10-year treatment benefit**	**No change in estimated benefit**	**302 (85.4%)**
	**Number with a change**	**52 (14.6%)**
	Range of change in benefit	-4 to 8%
	Mean change in benefit	-1.05%
	Median change in benefit	-1.0%
**15-year survival outcome**	**No change in predicted outcome**	**279 (78.8%)**
**(*n* = 354)**		
	**Number with a change**	**75 (21.2%)**
	Range of change in survival	-27% to 9%
	Mean change in survival	-2.4%
	Median change in survival	-2%
**15-year treatment benefit**	**No change in estimated benefit**	**288 (81.3%)**
	**Number with a change**	**66 (18.7%)**
	Range of change in benefit	-4 to 10%
	Mean change in benefit	1.1%
	Median change in benefit	1.0%

## Discussion

The introduction of MRI has undoubtedly been one of the most significant changes in prostate cancer diagnostics in decades [2]. A number of groups are now also exploring using MRI in screening trials although at this juncture it is unclear if MRI will pass the Wilson and Jungner criteria to reduce mortality or perhaps only in helping reduce the harms of screening [27, 28]. At a more immediate level MRI is being proposed as a useful adjunct to risk stratification in decision making albeit with surrogate markers of outcome such as adverse pathology, biochemical relapse free survival or progression rates on Active Surveillance [29–32]. In contrast there is uncertainty on whether MRI will actually improve long-term survival outcomes [10, 13, 33].Modelling studies so far have found little evidence for this and MRI may in fact contribute to over-diagnosis and over-treatment [10].

MRI might provide 3 routes to better prognostics (i) more accurate staging (ii) more accurate biopsy sampling and (iii) independent value derived from MRI lesion conspicuity (PI-RADS/Likert scores). Of these (i) and (ii) lend themselves to immediate assessment. In a head to head comparison MRI was found to be superior to nomograms in predicting extracapsular extension at surgery which is perhaps not surprising given its ability to visualise the tumours pre-surgery [34]. Jansen et al.2019 on the other hand found only a small advantage in adding MRI to currently used pre-surgical nomograms [31]. There is less data for a benefit of this better staging in longer-term outcomes. One study used retrospective MRI features (lesion presence and staging but *not* lesion conspicuity) to show added value to EAU and CAPRA scores in predicting metastasis and PCM after radical treatment [30]. The majority of these studies recruited men from screened or high penetrance PSA tested populations and unsurprisingly detected cancers were often not palpable clinically. In most randomised trials of detection of cancers comparing with and without MRI, T stage is either not mentioned or limited to ≤ T2 disease hence including a bias in selection for earlier and smaller tumours [5–8]. In the present study, which did not have exclusions and hence a wider spectrum of disease at presentation, we found that MRI stage shift compared to digital rectal examination was the biggest contributor to a change in overall pre-treatment risk grouping but still only occurred in about 8% of cases. Studies looking at the concordance between biopsy and radical prostatectomy histology have found conflicting results on the value of targeted versus systematic biopsies [35, 36]. In general, the consensus seems to be that the combination of the two provides the best concordance with final surgical pathology [35–37]. Much less is known about the added value of MRI lesion features (PI-RADS/Likert) as an independent prognostic or predictor of outcome. Lesions visibility is being explored by different groups with so far inconclusive results [38, 39]. In a review of the literature Stabile et al.found 6 studies on the predictive role of the PI-RADS score and concluded that reliable evidence was limited but there was a trend for better prediction of biochemical relapse after surgery [40]. There is no current data on impact on overall prognosis and survival. Much of this debate is confounded by what the term “significant cancer” means. What may be significant for diagnosis may not be equally as important in prognosis particularly when only histologic grade is considered. For example, GG2 in 5% of biopsy samples, PSA 5 ng/ml and T1 in a 78-year-old is diagnostically significant but unlikely to have any overall treatment survival impact.

To our knowledge ours is the first study to attempt to model the impact of reallocations by MRI on both stage and grade group changes in pre-treatment risk and prognostic group performance. Previous studies have often compared stage only i.e. between digital rectal examination with MRI and also confined their work in men undergoing radical prostatectomy [41, 42]. This we believe introduces a cohort selection bias in that these men often have lower disease burdens and are less likely to have clinically palpable tumours. In our series the rate of stage and grade changes in the diagnostic information were not as marked as these other papers and likely represent the fact that our cohort included men who present with more significant disease overall (typical of unscreened cohorts seen in the UK and elsewhere in the world). As an example, only 9% of the men in the paper by Soeterik et al. had clinical T3 disease whereas the rate was 28.9% in our cohort [42]. Thus, we believe our work is more representative of a real-world diagnostic cohort of men. In this context, although only a simulation, our work in pre-treatment men does suggest that more granular risk-tier systems have better performance for PCM estimations. Risk and prognostic modelling should also in the future be moving to more individualised prognostics [25]. In this regard we found that MRI had less impact on individual OS and treatment benefit estimations. Robust studies to assess MRI impact on OS will be complex to undertake given the number of competing variables to consider, and the requirement for large cohorts with a long-term follow up. Although a few are underway they propose to include relatively small numbers and It will be sometime before any meaningful survival outcomes are measurable [43]. It is more likely that population-based studies will report sooner if it can be reliably inferred as to whether staging was done by MRI or clinical means.

Our study has limitations being a single centre retrospective report with a small number of patients. Scanning was performed predominantly at 3.0 T and reported by experienced radiologists, and therefore may not be reflective of the practice in all healthcare settings. We also did not compare for any differences between readers given the high levels of experience of our uro-radiology team. Our cohort was also drawn from an unscreened population and we did not exclude based on PSA, stage or age hence our men are likely to represent a more mixed “group than studies which have only considered men who went onto radical prostatectomy. We also excluded men where we could not get intact data for both clinical and MRI staging/biopsies and cannot comment on men who may have had a negative MRI after clinical assessment and did not proceed to a biopsy. These factors may have introduced a degree of selection bias. Although we have made a distinction between systematic and targeted biopsies, we cannot rule out operator-bias in sample collection who were not blinded to the MRI report (e.g. systematic biopsies may potentially have included a target area). In some cases, the MRI was available before the clinic examination and may have influenced interpretation of the digital rectal examination (in 89/370, 24%). We did not however find any significant differences in the proportions with a stage change between those who did or did not have MRI before clinical examination (data not shown). We extrapolated the differences seen between contemporary clinical based versus MRI based characterisation onto a historic cohort diagnosed up to 20 years ago, hence there may be other differences in baseline characteristic that affected the outcomes. MRI quality and radiologist experience are variable and may have a direct impact on outcomes, in the future computer-aided diagnosis (CAD) systems may help reduce this variability, initial studies have shown promise in this area. However, these have demonstrated methodological limitations and biases which future studies will need to address to ensure generalisability [44]. Finally, all the risk models we tested were developed prior to the era of pre-biopsy MRI and we have assumed a similar proportion of deaths for the new simulated risk groups which may therefore be under or over estimates. As such we could not reliably do statistical comparison on simulated data to look for differences in performance hence our derived C indices are considered descriptive only. We further acknowledge that there are many other risk models which we have not tested in this study e.g. CAPRA score, NCCN 6-tier model [45]. Our results are therefore best interpreted as hypothesis generating rather than confirmatory. Positive attributes include the fact that all men had high quality MRI and targeted biopsies in a well-established diagnostic service.

In summary, we explored the potential impact of MRI on risk and prognostic model performance when based on diagnostic information before a treatment decision is made or administered. We found that more granular tiered models may be better to use in the MRI era and less susceptible to changes in risk assignments when based on diagnostic clinico-pathological features. The impact on use of individualised prognostic system may be even less. These results require further validation in larger cohorts including both screened and unscreened populations as well as testing with other risk model systems. Nevertheless, this work may give confidence that modern prognostic groups can continue to be used in the MRI diagnostic era for risk stratified decision making and patient counselling until such time as long-term survival data is available and head to head model comparison studies can be made.

## Supplementary Information

**Additional file 1:** 

## Data Availability

The datasets used and/or analysed during the current study available from the corresponding author on reasonable request.
