# Supplementary material for: Assessing the impact of MRI based diagnostics on pre-treatment disease classification and prognostic model performance in men diagnosed with new prostate cancer from an unscreened population
Source: BMC Cancer. 2022 Aug 11;22:878. doi: 10.1186/s12885-022-09955-w (PMC9367076; doi:10.1186/s12885-022-09955-w)
Supplement: Supplementary file 1 — Additional file 1: [file 12885_2022_9955_MOESM1_ESM.docx]

**Supplementary Methods M1**

**Illustration of the method to simulate the MRI adjusted changes in risk group performance in prediction of prostate cancer mortality (PCM) (CPG model as an example)**

**Step 1.** The percentage difference from the clinical and MRI data in this present study was computed. This exercise helped the forecast of percentage that would change if MRI was introduced.

| **CPG original distribution previous**  **study (ref 24)** | **Freq.** | **Percent** | **% diff derived from clinical VS MRI in this study** | **New % required for simulated image-based CPG** |
| --- | --- | --- | --- | --- |
| 1 | 1,740 | 17.16 | -1.1 | 16.0 |
| 2 | 2,078 | 20.5 | -3.2 | 17.3 |
| 3 | 1,637 | 16.15 | -1.9 | 14.3 |
| 4 | 3,052 | 30.1 | 4.3 | 34.4 |
| 5 | 1,632 | 16.1 | 1.9 | 18.0 |
| Total | 10,139 | 100 |  | 100.0 |

**Step 2**. The percentage difference from step 1 was then applied to the original east of England 10,139 dataset to derive a new cohort distribution. Table 2 shows number and percentage after applied % from step 1.

| **Simulated image-based CPG** | **Freq.** | **Percent** |
| --- | --- | --- |
| 1 | 1,627 | 16.05 |
| 2 | 1,753 | 17.29 |
| 3 | 1,451 | 14.31 |
| 4 | 3,483 | 34.35 |
| 5 | 1,825 | 18.00 |
| Total | 10,139 | 100.00 |

**Step 3**. We next randomly assigned alive or deceased status to each CPG (using the *statarand* command, a Stata randomisation module) based on % distribution of deceased /alive derived from the original east of England 10,139 dataset (ref 24)

The same as above was done for the EAU and AUA risk model simulations.
